# Co-Fermentation of Glucose–Xylose Mixtures from Agroindustrial Residues by Ethanologenic *Escherichia coli*: A Study on the Lack of Carbon Catabolite Repression in Strain MS04

**DOI:** 10.3390/molecules27248941

**Published:** 2022-12-15

**Authors:** Estefanía Sierra-Ibarra, Alejandra Vargas-Tah, Cessna L. Moss-Acosta, Berenice Trujillo-Martínez, Eliseo R. Molina-Vázquez, Alberto Rosas-Aburto, Ángeles Valdivia-López, Martín G. Hernández-Luna, Eduardo Vivaldo-Lima, Alfredo Martínez

**Affiliations:** 1 Departamento de Ingeniería Celular y Biocatálisis, Instituto de Biotecnología, Universidad Nacional Autónoma de Mexico. Av. Universidad 2001, Col. Chamilpa, Cuernavaca 62210, Mexico; 2 Departamento de Ingeniería Química, Facultad de Química, Universidad Nacional Autónoma de Mexico, Ciudad de Mexico 04510, Mexico

**Keywords:** lignocellulosic hydrolysates, *Escherichia coli*, bioethanol, monosaccharides co-consumption, catabolite repression

## Abstract

The production of biofuels, such as bioethanol from lignocellulosic biomass, is an important task within the sustainable energy concept. Understanding the metabolism of ethanologenic microorganisms for the consumption of sugar mixtures contained in lignocellulosic hydrolysates could allow the improvement of the fermentation process. In this study, the ethanologenic strain *Escherichia coli* MS04 was used to ferment hydrolysates from five different lignocellulosic agroindustrial wastes, which contained different glucose and xylose concentrations. The volumetric rates of glucose and xylose consumption and ethanol production depend on the initial concentration of glucose and xylose, concentrations of inhibitors, and the positive effect of acetate in the fermentation to ethanol. Ethanol yields above 80% and productivities up to 1.85 g_EtOH_/Lh were obtained. Furthermore, in all evaluations, a simultaneous co-consumption of glucose and xylose was observed. The effect of deleting the *xyIR* regulator was studied, concluding that it plays an important role in the metabolism of monosaccharides and in xylose consumption. Moreover, the importance of acetate was confirmed for the ethanologenic strain, showing the positive effect of acetate on the co-consumption rates of glucose and xylose in cultivation media and hydrolysates containing sugar mixtures.

## 1. Introduction

Ethanol is an important commodity in transportation and industry, as it is considered a sustainable, renewable and eco-friendly energy source [1]. Unlike oil-derived fossil fuels, ethanol can be produced from renewable lignocellulosic biomass through microbial fermentation; particularly, the synthesis of ethanol using slurries from agroindustrial residues as culture media has additional economic and environmental benefits [2]. Therefore, several methodologies have been applied for the pretreatment of these feedstocks to obtain slurries enriched with fermentable sugars [3]. It is important to note that lignocellulose has three main components: cellulose, hemicellulose, and lignin. These components are arranged into macrofibrils that confer structural stability to the plant cell wall [4]. When these fibrils are pretreated with diluted acid, they release syrups containing fermentable sugars, mainly glucose and xylose. 

Lignocellulose is found in agricultural waste, which is considered a major global issue as it is underutilized and produced in huge quantities, making it a contaminant of difficult disposal. Latin American countries, such as Mexico and Colombia, are known for their variety of agricultural and timber products, making them major producers of agricultural waste [5]. In Mexico, the production of tequila and mezcal generates more than 360,000 metric tons of agave bagasse per year [6]. Otherwise, 42 million tons of corn stover are generated each year [7], making these residues some of the most relevant in the country, as they have high potential to be used as feedstock in the synthesis of value-added products. Timber is another important industry in Mexico. For example, the production of teak wood constitutes 29,000 ha of planted trees around the country (https://www.eleconomista.com.mx/opinion/Plantaciones-forestales-comerciales-de-teca-en-Mexico-II-20190110-0137.html (accessed on 20 October 2022)); thus, the residues that this industry produces in Mexico are also significant. Meanwhile, Colombia is one of the most important producers of coffee in the world [8], where spent coffee grounds constitute the main byproduct, with potential to be converted into ethanol, biodiesel and other high-value biorefinery products [9]. Finally, in recent decades, the production of barley has increased to approximately 140 million tons per year due to the rise in craft breweries and its use for animal and human consumption [10], making the development of processes for its waste valorization an attractive venture. 

The residues from the agroindustrial crops mentioned above contain different concentrations of fermentable sugars. Nevertheless, their use as feedstocks to produce value-added products such as ethanol represents an important economic and environmental opportunity for the mentioned countries. Hence, the study of ethanologenic fermentation of hydrolysates obtained under different treatment conditions and with different concentrations of sugars and inhibitors is relevant for the development and improvement of specific production processes for each agricultural waste. However, wild-type ethanologenic microorganisms are not able to efficiently ferment all the sugars contained in the hydrolysate slurries, which limits the use of lignocellulosic residues for ethanol production [11]. Therefore, metabolically engineered ethanologenic *Escherichia coli* strains that produce ethanol from glucose or xylose have previously been developed. These strains achieved yields of up to 90% when cultured in mineral media or in certain lignocellulosic hydrolysates [7]. Furthermore, unlike other common ethanologenic microorganisms such as yeast, *E. coli* can consume different hexoses and pentoses such as glucose, mannose and galactose, and xylose and arabinose. Nonetheless, *E. coli* preferentially consumes glucose over other sugars; therefore, the fermentation of pentoses, such as xylose, becomes inefficient and is often not completed [12]. The preference in glucose consumption is due to the phosphoenolpyruvate-dependent glucose phosphotransferase system (PTS) [13]. This system is involved in the regulation of several cellular processes, such as carbon catabolite repression, and is part of a global regulatory network controlling the capability of cells to find, select, transport and metabolize several types of carbon sources [14].

On the other hand, the transport and metabolism of xylose in *E. coli* are mediated by two major transcriptional units, *xylFGHR* and *xylAB*, whose expression is governed by promoters activated by xylose and repressed by glucose [15]. However, the xylose operon regulatory protein *xylR* has a weak non-sugar regulated promoter [16]. Moreover, it has been shown that in the presence of xylose, *xylR* forms a dimmer that activates transcription of the transcriptional units mentioned above [17]. It has also been reported that in wild-type and ethanologenic strains of *E. coli*, two-point mutations on the *xylR* gene release the carbon catabolic repression of glucose and arabinose over xylose, since they provide a higher binding affinity to the DNA of promoter regions for the xylose catabolic operons [16].

In this work, the metabolic engineered *E. coli* strain MS04 was used for ethanologenic fermentation of lignocellulosic syrups from different agricultural wastes. Fermentations in simulated hydrolysates were also performed as controls. Co-fermentation of hexoses and pentoses, glucose and xylose, as well as ethanol production, were evaluated in *E. coli* MS04 and a mutant of this strain. This work discusses the simultaneous consumption of glucose and xylose by *E. coli* MS04 in different lignocellulosic hydrolysates and mineral media, while also analyzing potential causes for the lack of catabolite repression in the strain.

## 2. Results and Discussion

### 2.1. Co-Consumption of Glucose and Xylose by E. coli MS04 in Simulated Media and Plant Hydrolysates Containing High Glucose Concentration

**Figure 1:**
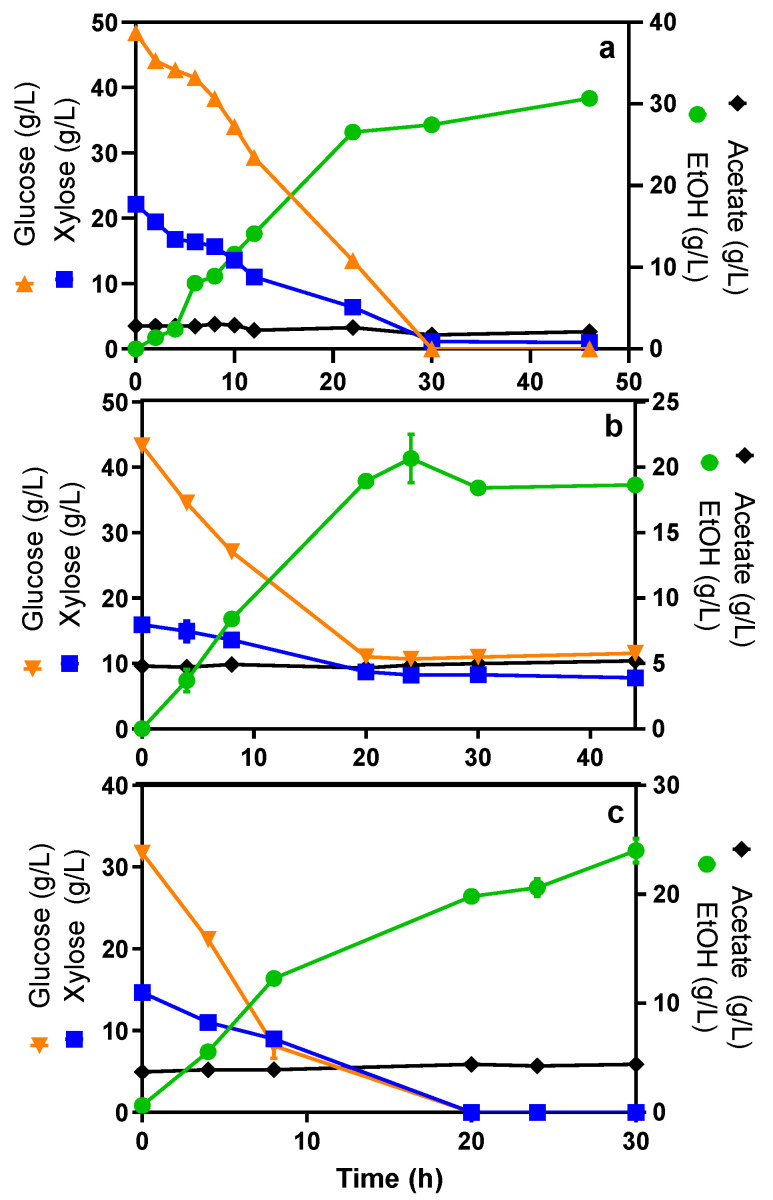
Glucose and xylose consumption and ethanol production by *E. coli* MS04 in LSH and ALH with syrups containing relatively high glucose concentrations and lower amounts of xylose. (**a**) Mineral medium, (**b**) teak wood, (**c**) agave bagasse.

*E. coli* MS04 was used for ethanol production in laboratory-simulated hydrolysates (LSH) with the so-called AM1 mineral medium (see Section 3) supplemented with glucose and xylose (Figure 1a), as well as agroindustrial lignocellulosic hydrolysates (ALH), such as teak wood residues (Figure 1b) and agave bagasse (Figure 1c). Given that enzymatic saccharification was performed after pretreatment and before fermentation, the glucose concentration of the slurries was higher than that of xylose. Kinetic and stoichiometric parameters are shown in Table 1.

Although both ALH and LSH have different sugar concentrations, it is interesting to note that both sugars were consumed simultaneously. In all the evaluated hydrolysates, most of the sugars were consumed between 5 and 20 h after the start of the experiments (Figure 1b,c). Depending on the hydrolysate, the volumetric consumption rate of glucose (Q_glc_) was 1.5 to 4 times higher than the consumption rate for xylose (Q_xyl_). In addition, the volumetric productivity of ethanol (Q_EtOH_) also shows a broad range of values (Table 1). Therefore, the variations in volumetric consumption and production rates appear to be determined by the nature of the residue and the initial concentration of fermentable monosaccharides.

The fermentation of the teak wood hydrolysate showed the lowest volumetric sugar consumption rates, as 25% and 53% of the initial glucose and xylose, respectively, remained at the end of the tests (Figure 1b). These low sugar consumption and ethanol productivity rates were due to the relatively high amounts of phenolic-derived compounds such as vanillin, vanillic acid, guaiacol and catechol [18] contained in these slurries (12 g/L), which are toxic to *E. coli* in concentrations above 1 g/L [19]. On the other hand, agave bagasse hydrolysates have been previously fermented into ethanol by yeasts, achieving productivities between 0.5 and 7.2 g_EtOH_/Lh [20,21]; however, in such studies, there were low consumption rates of pentoses. Similar results were reported for the fermentation of pine hydrolysates amended with yeast extract and fermented with the ethanologenic *E. coli* KO11, where a Q_p_ of 0.73 g/Lh was achieved without a complete xylose depletion [22]. Meanwhile, the use of ethanologenic *E. coli* MS04 in agave bagasse hydrolysate has shown yields and productivities of 81.6–85.3% and 0.68–1.2 g_EtOH_/Lh for treatments with ionic liquids and organosolv, respectively [23]. These values are in accordance with those obtained in the present work (Table 1). Additionally, in both studies, a simultaneous consumption of glucose and xylose was observed, which in turn enhanced the efficiency of the fermentation process.

As shown in Figure 1, despite the differences in biomass treatment conditions and the varying contents of glucose and xylose in the hydrolysates, the co-consumption of hexoses and pentoses was noteworthy in all three experiments. Furthermore, in the case of teak wood hydrolysates, the higher amount of available glucose at the initial fermentation times and the content of inhibitory amounts of phenolic compounds did not seriously hinder the consumption of sugars.

### 2.2. Co-Consumption of Glucose and Xylose by E. coli MS04 in Simulated and Plant Hydrolysates Containing High Xylose Concentration

**Figure 2:**
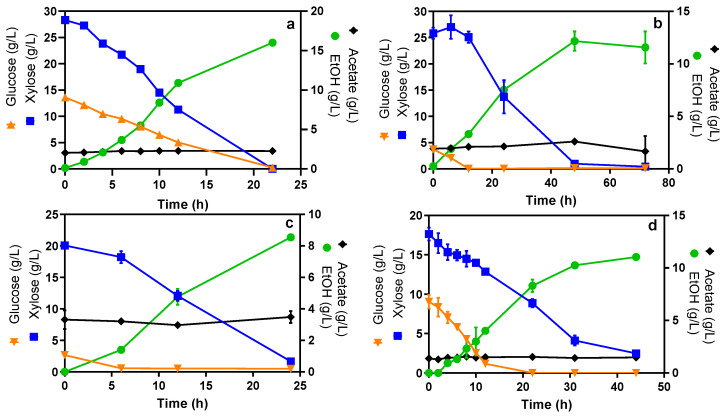
Glucose and xylose consumption and ethanol production by *E. coli* MS04 in LSH and ALH with higher proportions of xylose. (**a**) Mineral medium, (**b**) corn stover, (**c**) barley straw, (**d**) spent coffee grounds.

Low-glucose-concentration syrups obtained from the thermochemical hydrolysis of corn stover (Figure 2b), barley straw (Figure 2c) and spent coffee grounds (Figure 2d) were used as media for ethanol production with *E. coli* MS04. It is important to note that, except for the one pertaining to the coffee grounds, the used slurries were not saccharified before the fermentation process. These results show that the simultaneous consumption of both monosaccharides was also observed when using lignocellulosic slurries with higher xylose concentrations.

Regarding spent coffee grounds (Figure 2d), this biomass has been used as feedstock for biodiesel production due to its chemical composition and desirable lipid profile [24]; however, the relatively high proportion of carbohydrates contained in this residue (approximately 13% and 42% of cellulose and hemicellulose, respectively) also makes it a suitable source for bioethanol production [9]. For example, in previous studies, ethanol production from spent coffee grounds hydrolysates was performed by using the yeast *S. cerevisiae* as a biocatalyst, achieving Y_EtOH_ and Q_EtOH_ up to 91% and 1.0 g/Lh, respectively [25], from the consumption of glucose. This productivity rate is on average 2.6 times higher than that reported in our study (Table 2). Nevertheless, an advantage of using *E. coli* MS04 as an ethanol-producing microorganism is that it allows the use of hexoses and pentoses as substrate. Furthermore, prior to fermentation, an extraction of oils and phenolic lignin derivatives from the spent coffee ground hydrolysates could be a suitable strategy to improve ethanol productivity in cultures with *E. coli* [26].

Fermentation of hydrolysates from corn stover (Figure 2b) and barley straw (Figure 2c) did not show a clear co-consumption of both monosaccharides because their initial glucose concentrations were under 4 g/L. This issue makes the analysis difficult because, as reported in previous studies, under limited-oxygen conditions, low glucose concentrations trigger high transcriptional levels of *ptsG*, a gene that is directly involved in catabolic repression, in turn affecting metabolic cellular regulation [27]. In addition, the glucose uptake rate is limited by glucose concentration [28], which explains the lower Q_glc_ for these syrups (Table 2). Meanwhile, similarly to sugar consumption rates, Q_EtOH_ seems to be negatively affected by the initial sugar concentration, being 0.5 to 4 times lower than other reports for ethanol production from corn and barley [29,30]. Furthermore, a Y_EtOH_ above 80% demonstrates that low glucose concentration affects the kinetics of the process but not the yields.

As expected, xylose uptake rates were higher than glucose rates in media with low glucose concentrations (Table 2) since sugar consumption is less regulated in these cases [29]. The only exception to this is the case of coffee grounds, where Q_glc_ was higher than Q_xyl_ (Table 2). From these results, we can infer that the kinetics of sugar consumption and ethanol production not only depend on the proportion of sugars, but also on the hydrolysate composition. This statement is reinforced when comparing the results for control cultures in mineral media (Table 1 and Table 2), where the cell growth expressed as X_max_ and µ is similar, and Q_EtOH_ is only slightly lower for the medium with more xylose, despite the differences in sugar consumption rates.

Taking into consideration the previously discussed results, this work aims to gain further insight into what causes the co-consumption of xylose and glucose with the ethanologenic *E. coli* MS04.

### 2.3. The Lack of Acetate in the Mineral Media Negatively Affects Sugar Consumption

One of the most studied inhibitory compounds contained in ALH is acetate, which is released during hemicellulose hydrolysis in concentrations between 1.5 and 13 g/L [31]. Nevertheless, some ethanologenic *E. coli* strains have shown tolerance and improved growth under certain concentrations of acetate; specifically, *E. coli* strain MS04 grows at higher rates when at least 2 g/L of acetate is present in the culture medium, and it is able to tolerate acetate concentrations of up to 10 g/L [32]. In addition, *E. coli* can co-consume mixtures of glucose, arabinose and xylose at a higher rate in the presence of acetate than when it is absent [33]. Accordingly, we studied the co-consumption of these three monosaccharides (at initial concentrations of 25 g/L of each sugar) and ethanol production by *E. coli* MS04 in mineral media without acetate (WOA) and with acetate (WA) at an initial concentration of 2 g/L.

**Figure 3:**
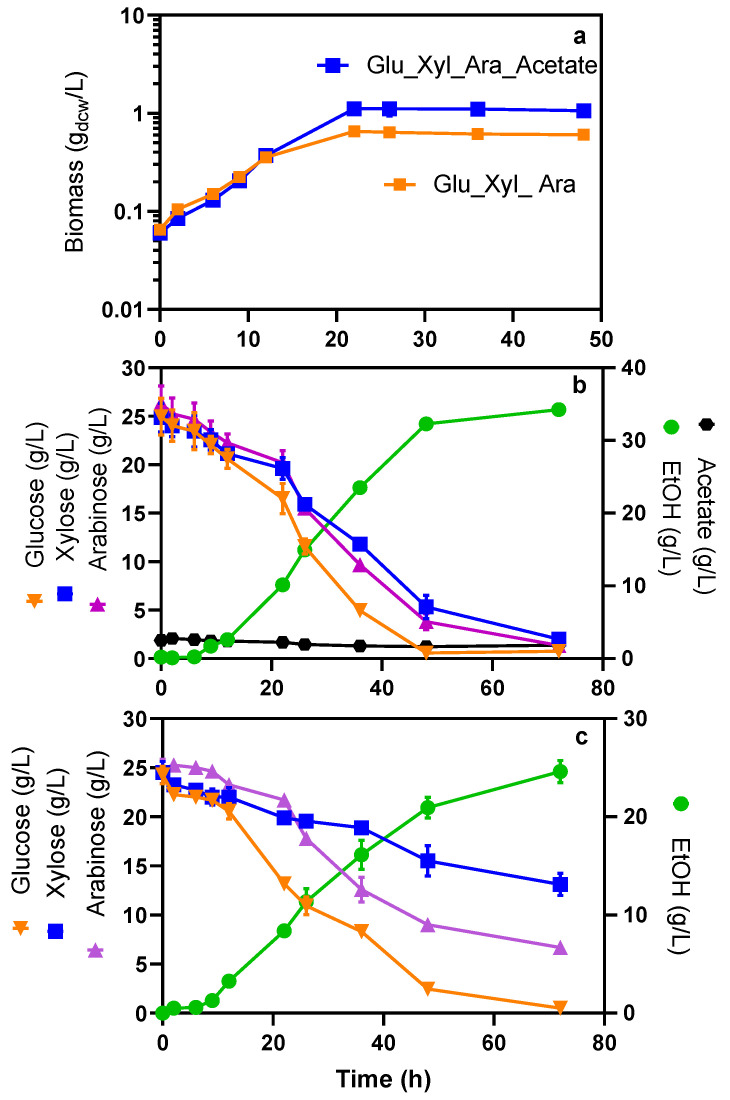
Kinetics of cell growth (**a**); glucose, xylose and arabinose consumption (25 g/L each at initial time), and ethanol production by *E. coli* MS04 in mineral media WA 2 g/L (**b**) and WOA (**c**).

The curves in Figure 3b,c show the positive effect that acetate has on the rates of sugar consumption, ethanol production and in maximum cell concentration (Table 3). In medium WA, glucose was completely consumed after 48 h, with minor amounts of xylose and arabinose remaining after this elapsed fermentation time, but these pentoses were completely consumed after 72 h (Figure 3b). Although the glucose consumption rate was 50% higher than the xylose and arabinose consumption rate (Table 3), it is important to note that there was simultaneous uptake of the three sugars and that a high ethanol yield was observed. On the contrary, the fermentation in the medium WOA showed lower and more variable sugar consumption (Figure 3c). Therefore, after 48 h, 6%, 39% and 22% of the initial concentrations of glucose, xylose and arabinose remained in the culture media, respectively. In addition, Q_EtOH_ and X_max_ (Table 3) were also 94% and 72% lower compared to the medium WA. Regarding µ values, the rate of cell growth does not seem to be affected by the presence of acetate.

The results described above demonstrate that under non-aerated conditions, moderate concentrations of acetate improve the consumption of sugars and the production of ethanol by *E. coli* MS04. This is due in part to the strain’s necessity for acetyl CoA generation as the *pflB* gene was deleted [32].

In relation to the simultaneous consumption of sugar mixtures, the curves in Figure 3b,c show that a lack of acetate does not release catabolic repression. Furthermore, the established hierarchy for sugars preferably consumed by *E. coli* is clearly noticeable in the fermentation WOA, where glucose was consumed at the highest rate, followed by arabinose and then xylose (Table 3). These results concur with the model of carbon catabolite repression in *E. coli*, which states that glucose is preferentially consumed, followed by arabinose and finally xylose. This is because the arabinose operon regulates xylose metabolism, leading to a favorable consumption of arabinose over xylose [34]. This behavior suggests that the acetate contained in the evaluated hydrolysates could be partly responsible for the lack of regulation of sugar uptake. Still, other regulatory mechanisms induce this phenomenon, making the consumption of sugars by *E. coli* MS04 strain a complex metabolic regulation system.

### 2.4. Xylose Consumption Is Governed by the XylR Activator 

**Figure 4:**
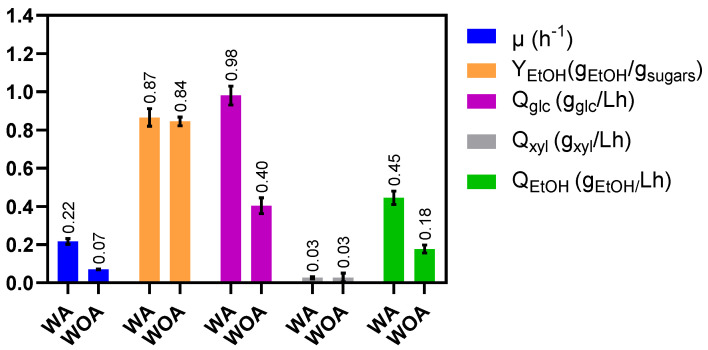
Ethanol yield and kinetic parameters for fermentation of 25 g/L (each) glucose-xylose mixtures by *E. coli* MS04 Δ*xylR* in mineral media with acetate (WA, 5 g/L) and without acetate (WOA) (bars indicate standard deviation from triplicate experiments).

In *E. coli*, XylR is a transcriptional activator for xylose uptake. Therefore, in the presence of xylose, it promotes the transcription of the *xylFGH* and *xylAB* operons, which are required for xylose metabolism [17]. Although *xylFGH* was deleted from *E. coli* MS04 strain genome, previous studies performed by our group have demonstrated that the MS04 parental strain also internalizes xylose through the galactitol transporter GatC while *xylAB* genes are transcriptionally active [35]. Hence, we studied the function of *xylR* in the co-utilization of sugars by *E. coli* MS04, its possible relationship to the lack of carbon catabolite repression and the combined effect with acetate. To this end, the *xylR* gene was deleted from the *E. coli* MS04 genome (*E. coli* MS04 Δ*xylR*) and fermentations were performed with this new strain. In these tests, glucose–xylose mixtures at initial concentrations of 25 g/L for each were fermented in two different mediums, one with 5 g/L of acetate and another without acetate. Figure 4 shows the ethanol yield and kinetic parameters obtained for these fermentations.

These results show that xylose consumption was null in both cultures, demonstrating the need of XylR for xylose metabolism in *E. coli*. Unlike MS04 (Table 3), the µ for the MS04 Δ*xylR* strain (Figure 4) was three times lower when there was no acetate in the culture medium, demonstrating the importance of xylose consumption for cell growth even in media containing glucose. Although Y_EtOH_ values were similar, glucose consumption and ethanol production rates were about 2.5 times lower for the fermentations WOA. Additionally, Q_glc_ in the presence of acetate was double compared to that observed for MS04 (Table 3 and Figure 4), suggesting that in the absence of an active xylose metabolism pathway, all the catabolic energy is directed towards glucose consumption.

The results obtained for *E. coli* MS04 Δ*xylR* demonstrate that, independently of the sugars consumed, *XylR* plays an essential role in xylose metabolism, and also highlight the importance of acetate for sugar consumption and ethanol production rates. Nevertheless, these results do not explain the lack of carbon catabolite repression in *E. coli* MS04. Therefore, an exhaustive review of the sequence of MS04 strain genome was performed, with the objective of finding possible gene deletions, changes or single-nucleotide polymorphism (a variation at a single position in a DNA sequence in the genome) that could help explain the co-utilization of sugars. Moreover, the sequence of *xylR* was carefully analyzed, as it has previously been reported that single-nucleotide polymorphisms in this gene void the preference for glucose over other monosaccharides [36]. Nevertheless, no changes were found on the MS04 *xylR* sequence. On the other hand, the results presented in this work show a clear co-fermentation of sugar mixtures under different media composition and lignocellulosic hydrolysates; therefore, additional studies are needed to reveal the causes for the lack of carbon catabolite repression in *E. coli* MS04.

## 3. Materials and Methods

### 3.1. Strains

#### 3.1.1. *Escherichia coli* MS04

*Escherichia coli* strain MS04 (MG1655: Δ*pflB*, Δ*adhE*, Δ*frdA*, Δ*xylFGH*, *gatC*-S184L, Δ*midarpA*, Δreg 27.3 kb, Δ*ldhA*) [7] was used as a biocatalyst for ethanol production in mineral medium and lignocellulosic hydrolysates.

#### 3.1.2. *Escherichia coli* MS04 ΔxylR

Deletion of *xylR* gene from *E. coli* MS04 was carried out using the phage transduction methodology. *Escherichia coli* strain MS04 is kanamycin (Km)-resistant due to a mutation in the FRT sequence. The Km-resistant cassette placed in the locus *xylFGH* was replaced by a chloramphenicol (Cm)-resistant cassette. This was performed by a P1 transduction event as previously reported [37]. An *E. coli* MG1655 strain Δ*xylFGH*::*Cm*^R^ constructed in our laboratory (unpublished data) was used as the donor strain. The mutant MS04 Δ*xylFGH*::*Cm*^R^ was selected in LB plates supplemented with Cm at 30 µg/mL, and Km sensitivity was verified. The cassette resistance replacement was confirmed by PCR. The resulting strain was used for a second P1 transduction process. Strain JW3541-2 (BW25113 Δ*xylR*::Km^R^) from the Keio collection was used as the donor strain [38]. Furthermore, the mutant MS04 Δ*xylFGH*::Cm^R^ Δ*xylR*::Km^R^ was selected in LB plates and supplemented with Cm and Km at 30 µg/mL each. Finally, the elimination of *xylR* gene in the MS04 background was confirmed by PCR.

### 3.2. Culture Media

#### 3.2.1. Laboratory-Simulated Hydrolysates

Control fermentations and the study of acetate effect with strain MS04, and cultures with MS04 Δ*xylR*, were performed in AM1 mineral medium [39]. The composition of the AM1 medium was 2.63 g/L (NH_4_)_2_HPO_4_, 0.87 g/L NH_4_H_2_PO_4_, 1 mL/L MgSO_4_7H_2_O (1 M), 1 mL/L KCl (2 M), 1 mL/L betaine HCl (1 M) and 1.5 mL/L trace elements. The medium was supplemented with 0.1 g/L sodium citrate and different concentrations of xylose, glucose and arabinose, and sodium acetate was added as needed. The trace element solution contains per liter 1.6 g FeCl_3_, 0.2 g CoCl_2_·6H_2_O, 0.1 g CuCl_2_, 0.2 g ZnCl_2_·4H_2_O, 0.2 g Na_2_MoO_4_, 0.05 g H_3_BO_3_ and 0.33 g MnCl_2_·4H_2_O. When required, 30 µg/L of Km or Cm was used for inoculum development.

#### 3.2.2. Lignocellulosic Hydrolysates

##### Teak Wood and Agave Bagasse Hydrolysates

Hydrolysates of teak wood and agave bagasse residues were prepared according to a previously reported methodology [40]. The procedure includes a thermochemical hydrolysis performed in a 5 L parr-type reactor containing a gas–liquid–solid system of 18% *w*/*w* biomass powder, 7% *w*/*w* SO_2_ and water. Typical reaction conditions were operated at 140 °C and 450 rpm for 90 min. The enzymatic saccharification of slurries obtained from thermochemical pretreatments of teak wood and agave bagasse was performed with a commercial cellulase cocktail (43 FPU g^−1^, 100 U_xylanase_ g^−1^) (NEO Biotech Co., Ltd., Xi’an, China) in a set of six 0.2 L (working volume) mini-reactors fitted with a peg mixer [6]. Enough sodium citrate was added to obtain a final concentration of 50 mM, while the slurry pH was adjusted to 4.8 by adding 10 N KOH, and 15 FPU/g_glucan_ of the cellulase cocktail was supplemented for saccharification.

##### Barley Straw and Corn Stover Hydrolysates

The hydrolysates from barley straw and corn stover were obtained through thermochemical hydrolysis carried out with a dry biomass charge of 15% *w*/*w*, 1% *w*/*w* H_2_SO_4_, and at 121 °C for 30 min (holding time). The hydrolysates were used as culture medium without additional pretreatment steps.

##### Spent Coffee Grounds Hydrolysates

Spent coffee grounds hydrolysates were obtained through a thermochemical pretreatment carried out with 15% *w*/*w* dry biomass, 1% *w*/*w* H_2_SO_4_, and at 121 °C for 30 min (holding time). After pretreatment, enzymatic saccharification was performed with commercial cellulase complex GC220 (Genencor International, Rochester, NY, USA) and β-glucosidase NS50010 (Novozymes, Copenhagen, Denmark) at 15 FPU/g_glucan_ and 30 UCB/g_dry biomass_, respectively. The saccharification process was performed at 50 °C and pH 4.5 for 48 h.

### 3.3. Fermentation in Simulated and Agroindustrial Lignocellulosic Hydrolysates 

Fermentations for both laboratory-simulated hydrolysates (LSH) and agroindustrial lignocellulosic hydrolysates (ALH) were carried out by using the ethanologenic *E. coli* strain MS04 [32] or its derivatives. The preinoculum was prepared by growing cells from a cryovial (1:1 glycerol 80% and strain at OD_600_ ~2.0 in mineral media) in test tubes containing 4 mL of mineral medium with 10 g/L of glucose or xylose. Afterwards, the cells were incubated for 12 h at 37 °C and 300 rpm. The inoculum was prepared by transferring the content of the test tubes to 200 mL fermenters containing AM1 mineral media, 20 g/L of glucose or xylose and 2 g/L of acetate. The inoculum was grown for ~24 h at 37 °C, 150 rpm and pH 7 (controlled with 2N KOH), until OD_600_ between 1.5 and 2 was reached. The cells were harvested by centrifugation (4 °C, 10 min, 4300× *g*), resuspended in fresh mineral media and then used to inoculate the fermenters with LSH or ALH at an initial OD_600_ ~0.5.

Fermentations were performed in the same bioreactor system used for saccharification. Before inoculation, the pH of the vessels containing ALH was adjusted to 7 with 8 N KOH and concentrated solutions of betaine (as osmoprotectant) to a final concentration of 1 mM. Then, phosphate buffer, citric acid and Km were added until final concentrations of 1 mM, 5 mM, 100 mg/L and 30 µg/L, respectively. The cultures were incubated at 37 °C, 150 rpm and pH 7 (controlled with 2 N KOH) until complete substrate depletion or consumption was achieved.

### 3.4. Biomass, Glucose, Xylose, Arabinose, Acetate and Ethanol Determinations

Growth in LSH was determined spectrophotometrically using an optical density of 600 nm (DU 70, Beckman Instruments, Inc. Fullerton, CA, USA). The values given by the spectrophotometer were converted to dry cell weight (DCW) by using a calibration curve, which indicates that 1 optical density at 600 nm = 0.37 g_DCW_/L. The samples were centrifuged, and the cell-free culture broth was frozen for its subsequent analysis. Xylose, arabinose and acetate concentrations were measured by high-performance liquid chromatography (Waters U6K, Millipore Co., Milford, MA, USA) using an Aminex HPX-87H ion exclusion column (300 mm × 7.8 mm; Bio-Rad Laboratories, Hercules, CA, USA) at 45 °C, while using 5.0 mM H_2_SO_4_ solution as the mobile phase (0.5 mL/min), a photodiode array detector at 210 nm (Model 996, Waters, Millipore Co) and a refractive index detector (Model 2410, Waters, Millipore Co., Milford, MA, USA). Glucose concentration in the culture medium was measured with a biochemical analyzer (YSI model 2700, YSI Inc., Yellow Springs, OH, USA). Finally, as described by Fernandez-Sandoval et al. [32], the ethanol produced from fermentations was quantified through gas chromatography (Agilent, 6850 series GC System, Wilmington, DE, USA).

### 3.5. Calculation of Kinetic and Stoichiometric Parameters

The kinetic and stoichiometric parameters for ethanol fermentations were calculated according to Equations (1)–(4) [19].

#### 3.5.1. Specific Growth Rate (µ)

In the case of experiments with hydrolysates, due to the syrups’ color and/or the presence of solids, the optical density was not measured. Thus, only the µ for control experiments was calculated during the exponential cell growth using Equation (1):

(1) $$µ=\frac{\ln\left(\frac{X}{X_{0}}\right)}{t-t_{0}}$$

where X (g_DCW_/L) is the biomass concentration at the end of exponential cell growth, X_0_ (g_DCW_/L) is the initial biomass concentration, t (h) is the time elapsed at the end of the exponential cell growth and t_0_ (h) is the initial time.

#### 3.5.2. Ethanol Yield (Y_EtOH_)

The ethanol yield from total sugars was calculated based on a theoretical yield of 0.51 g_EtOH_/g_sugar_ according to Equation (2).

(2) $$Y_{\mathrm{EtOH}}=\frac{{\mathrm{EtOH}}_{f}-{\mathrm{EtOH}}_{0}}{(S_{t0}-S_{\mathrm{tf}})\times 0.51}\times 100\%$$

where EtOH_f_ (g_EtOH_/L) is the maximum ethanol concentration, EtOH_0_ (g_EtOH_/L) is the initial ethanol concentration, S_T0_ (g_sugars_/L) is the sum of initial sugar concentration and S_to_ and S_tf_ (g_sugars_/L) are the sum of sugar concentration at the start and end of the fermentations.

#### 3.5.3. Volumetric Sugar Consumption Rate (Q_S_)

The volumetric consumption rates for individual sugars, i.e., glucose (glc), arabinose (ara) or xylose (xyl), were determined by Equation (3).

(3) $$Q_{S}=\frac{S_{0}-S_{f}}{t_{f}-t_{0}}$$

where S_0_ (g_sugar_/L) is the initial sugar (glc, ara or xyl) concentration, and S_f_ (g_sugar_/L) is the sugar concentration (glc, ara or xyl) at the end of the fermentation.

#### 3.5.4. Volumetric Productivity of Ethanol (Q_EtOH_)

The volumetric ethanol production rate was calculated using Equation (4).

(4) $$Q_{\mathrm{EtOH}}=\frac{{\mathrm{EtOH}}_{f}-{\mathrm{EtOH}}_{0}}{t_{\mathrm{EtOH}}-t_{0}}$$

where EtOH_f_ (g_EtOH_/L) is the maximum ethanol concentration, EtOH_0_ (g_EtOH_/L) is the initial ethanol concentration, t_EtOH_ is the elapsed time (h) at the maximum ethanol concentration and t_0_ is the initial time (h).

## 4. Conclusions

The ethanologenic *Escherichia coli* strain MS04 was able to efficiently produce ethanol from several lignocellulosic hydrolysates, which were obtained from different pretreatments and saccharification processes. Despite the differences in biomass treatment conditions and the varying contents of glucose and xylose, a clear co-fermentation of hexoses and pentoses in sugar mixtures under different media compositions and lignocellulosic hydrolysates was shown, indicating a partial lack of carbon catabolite repression. Even the presence of phenolic compounds that inhibit *E. coli* growth and fermentation did not seriously hinder the co-consumption of sugars and conversion into ethanol. The volumetric rates of sugar consumption and ethanol production depend on the proportion of initial glucose and xylose, concentrations of inhibitors and a positive effect of acetate (generated in the hydrolysates from the deacetylation of hemicellulose). Furthermore, the deletion of the *xylR* gene involved in xylose metabolism confirms its essential role in xylose consumption. These results give a first insight into the metabolism of monosaccharides in *E. coli* MS04 from a phenomenological perspective, but additional studies are needed to reveal the causes for the lack of carbon catabolite repression in *E. coli* MS04.

## Figures and Tables

**Table 1:** Ethanol yields and volumetric parameters for the fermentation of hydrolysates with relatively high glucose concentrations (values in parenthesis indicate standard error from duplicates).

Parameter	Control LSH	Teak Wood	Agave Bagasse
X_max_ (g_DCW_/L)	1.69	ND	ND
µ (h^−1^)	0.16	ND	ND
Y_EtOH_(%)	88	92 (5)	81 (5)
Q_glc_ (g_glu_/Lh)	1.61	0.72 (0.00)	1.59 (0.02)
Q_xyl_ (g_xyl_/Lh)	0.69	0.18 (0.02)	0.73 (0.01)
Q_EtOH_ (g_EtOH_/Lh)	0.92	0.42 (0.02)	0.96 (0.05)

Xmax: maximum cell mass concentration, DCW: dry cell weight, ND: not determined, µ: specific growth rate, Y_EtOH_: ethanol yield from consumed sugars; Q_glc_: volumetric consumption rate of glucose, Q_xyl_: volumetric consumption rate for xylose, Q_EtOH_: volumetric productivity of ethanol.

**Table 2:** Ethanol yields and volumetric parameters for the fermentation of hydrolysates with relatively high xylose concentrations (values in parenthesis indicate standard deviation from triplicates).

Parameter	Control LSH	Corn Stover	Barley Straw
X_max_ (g_DCW_/L)	1.61	ND	ND
µ (h^−1^)	0.19	ND	ND
Y_EtOH_(%)	75	81 (1)	82 (4)
Q_glc_ (g_glc_/Lh)	0.61	0.31 (0.01)	0.35 (0.02)
Q_xyl_ (g_xyl_/Lh)	1.29	0.52 (0.02)	0.77 (0.03)
Q_EtOH_ (g_ETOH_/Lh)	0.72	0.25 (0.02)	0.36 (0.01)

**Table 3:** Ethanol yields and volumetric parameters for fermentation of 25 g/L (each) glucose–xylose–arabinose mixtures by *E. coli* MS04 in mineral media WA 2 g/L and WOA (values in parenthesis indicate standard error from duplicates).

Parameter	WA	WOA
µ (h^−1^)	0.14 (0.00)	0.13 (0.00)
X_max_ (g_DCW_/L)	1.12 (0.09)	0.65 (0.06)
Y_EtOH_ (%)	95 (5)	90 (2)
Q_glc_ (g_glu_/Lh)	0.51 (0.04)	0.33 (0.01)
Q_xyl_ (g_xyl_/Lh)	0.32(0.02)	0.16 (0.00)
Q_ara_ (g_ara_/Lh)	0.35 (0.02)	0.25 (0.01)
Q_EtOH_ (g_ETOH_/Lh)	0.66 (0.01)	0.34 (0.02)

## Data Availability

The data presented in this study are available on request from the corresponding author.

## Sample Availability

Samples of the compounds are available from the authors.
